# Hybrid Drug Delivery System Designed from Spatiotemporal Hierarchical Controlled-Release Strategy Co-Delivering Rutin and Resveratrol for Coordinated Anti-Tumor Immunotherapy

**DOI:** 10.3390/pharmaceutics18070872

**Published:** 2026-07-16

**Authors:** Weinan Li, Sisi Yan, Yingying Gao, Yuhan Fu, Yutong Mei, Yanhong Wang, Zhixin Yang

**Affiliations:** 1 School of Pharmacy, Heilongjiang University of Chinese Medicine, Harbin 150040, China; 2 Key Laboratory of Basic and Application Research of Beiyao, Heilongjiang University of Chinese Medicine, Ministry of Education, Harbin 150040, China

**Keywords:** rutin, resveratrol, liposome-micelle hybrid delivery system, spatiotemporal hierarchical controlled, antitumor

## Abstract

**Background**: The highly heterogeneous and dynamically evolving tumor microenvironment leads to the development of drug resistance and recurrence in traditional therapies. Although immunotherapy demonstrates unique advantages, its clinical utility remains constrained by the suboptimal immunogenicity and the limited effect of monotherapy. Herein, a hybrid drug delivery system based on a spatiotemporal hierarchical controlled-release strategy was proposed to achieve dual immunotherapy with immune checkpoint blockade (ICB) and immunogenic cell death (ICD) to promote synergistic anti-tumor therapy. **Methods**: A liposome–micelle hybrid drug delivery system (RUT-RPP-LP) was constructed using a lipid bilayer composed of dioleoyl phosphatidylethanolamine/hemisuccinyl cholesterol to encapsulate rutin (RUT) and to form an inner cavity-encapsulated resveratrol micelle (RPP). RUT-RPP-LP was characterized, and its pH sensitivity and release behavior were investigated. Subsequently, a colon cancer tumor-bearing mouse model was constructed to evaluate the in vivo targeted anti-tumor effect and biological safety. On this basis, the combined mechanism of ICB and ICD was preliminarily explored. **Results**: RUT-RPP-LP possessed excellent formulation characteristics, stability, and biocompatibility, achieving graded controlled release of drugs via responding to the TME and lysosomal acidity, respectively. Obviously, RUT-RPP-LP could specifically target the tumor site, induce the occurrence of ICD, and simultaneously block the PD-1/PD-L1 immune checkpoint signaling pathway, thereby enhancing the function of T cells and inducing apoptosis of tumor cells. **Conclusions**: The RUT-RPP-LP based on the hierarchical controlled-release strategy exerted a spatiotemporally coordinated enhancement of anti-tumor immunity, and may provide a novel combinatorial approach to overcome the low response of immunotherapy in solid tumors.

## 1. Introduction

Malignant tumors represent one of the leading causes of global mortality. Their lethality stems not only from the uncontrolled proliferation of malignant cells, but also critically from the particularity of the tumor microenvironment (TME). The TME comprises disorganized vasculature, dense extracellular matrix, and immunosuppressive cells [1]. This multifaceted barrier impedes therapeutic drug delivery while concurrently fostering immune evasion, thereby dampening endogenous anti-tumor immunity [2,3,4]. Although highly specific for blocking a single pathway, traditional monotherapy cannot simultaneously eliminate the multi-dimensional immunosuppressive network in the TME and restore systemic immune competence, thereby leading to unsatisfactory clinical responses or distant metastasis of the tumor [5]. Accordingly, developing novel, rational, and multimodal therapeutic strategies that effectively remodel the TME and activate anti-tumor immunity has emerged as a central priority in contemporary anti-tumor therapy research.

The advent of immunotherapy has brought revolutionary progress to tumor treatment [6]. Immune checkpoint blockade (ICB), a representation of modern immunotherapy, significantly extends the survival time of some patients by relieving the inhibitory signals of T cells [7,8]. However, monotherapy with ICB faces bottlenecks such as low response rate, immune-related adverse reactions, and insufficient immunogenicity in clinical practice [9]. Studies have supported that the combination of ICB with immunogenic cell death (ICD) inducers elicits synergistic immunity through a cascade reaction [10]. ICD induction therapies (such as chemotherapeutics, radiotherapy, and photodynamic therapy) can trigger the release of damage-associated molecular patterns (DAMPs) from tumor cells, promoting the maturation and antigen presentation of dendritic cells (DCs). Concurrently, immune checkpoint inhibitors like programmed death-1/programmed death ligand-1 (PD-1/PD-L1) antibodies can reverse T cell exhaustion and enhance the tumor-killing ability of effector T cells [11,12,13]. Collectively, this dual-modality strategy provides a new direction for breaking through the limitations of single immunotherapy.

Rutin (RUT) and resveratrol (RES), representative polyphenolic compounds derived from two distinct classes of traditional Chinese herbs, exhibit unique potential in anti-tumor immune regulation [14,15]. Through in vitro and in vivo experiments, our research team has previously ascertained that RUT released into tumor tissues can suppress matrix metalloproteinase-9 (MMP9) to downregulate PD-L1 expression, thereby blocking the PD-1/PD-L1 immune checkpoint pathway and enhancing the immune clearance function of CD8+ T cells [16]. Simultaneously, in vitro cytological evaluations were conducted to confirm that RES micelles (RPP) were taken up by tumor cells and released RES through lysosomal escape, and then induced endoplasmic reticulum stress and reactive oxygen species (ROS) burst, resulting in calreticulin (CRT) exposure, high-mobility group box 1 (HMGB1) release, and DCs maturation, thereby triggering ICD in tumor cells and initiating systemic anti-tumor immune response [17]. Nevertheless, the clinical application of both agents remains hindered by defects in their physicochemical properties, including poor aqueous solubility and low bioavailability [18]. How to achieve efficient co-delivery of RUT and RES and precisely regulate their temporal and spatial release has become a key challenge to exerting their immune synergistic effect. Drug co-delivery systems, especially the liposome–micelle hybrid (LMH) delivery system, integrate the biocompatibility of liposomes (LPs) with the efficient drug-loading capacity of micelles, and can simultaneously encapsulate drugs of disparate properties and achieve targeted accumulation at the tumor site and stimulus-responsive drug release, which is conducive to exerting multi-mechanism anti-tumor synergistic treatment [19,20].

**Scheme 1:**
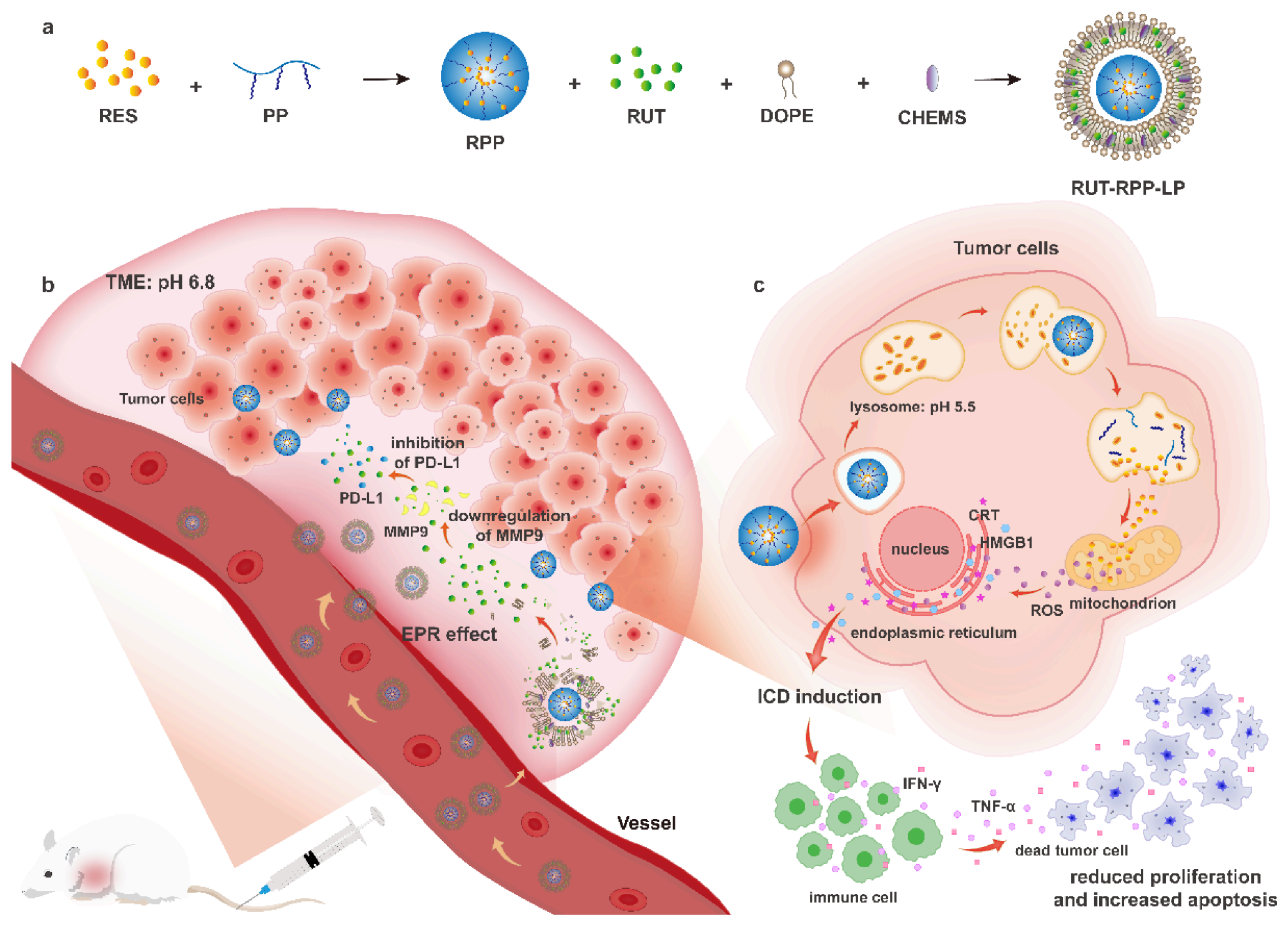
Schematic diagram of the construction of the dual-drug co-loading LMH delivery system RUT-RPP-LP (**a**), the release of RUT from the hybrid drug delivery system in response to TME to block the immune checkpoint PD-1/PD-L1 pathway (**b**), and the release of RES from RPP in response to the acidic lysosomal environment to induce ICD (**c**).

Facing the limitations of tumor monotherapy and the application of free Chinese herb active ingredients, based on our previous research findings, this study creatively constructed a pH-responsive dual-drug co-loading hybrid delivery system, RUT-RPP-LP, to achieve the synergistic treatment of ICB and ICD in malignant tumors. This system exploits the enhanced permeability and retention effect (EPR) for passive tumor targeting and enables a spatiotemporal hierarchical controlled-release strategy grounded in different acidity environments inside and outside the tumor cell. Specifically, in the weakly acidic TME (pH 6.8) environment, RUT and RPP are first released, which downregulates PD-L1 expression and then inhibits the PD-1/PD-L1 immune checkpoint. Meanwhile, the RPPs are internalized by tumor cells, undergo protonation and disintegration within acidic lysosomes (pH 5.5), mediate lysosomal escape, and elicit ICD (Scheme 1). RUT-RPP-LP overcomes inherent pharmaceutical limitations associated with free RUT and RES. Critically, its therapeutic effect is superior to that of single-agent delivery by concurrently engaging ICB and ICD pathways to achieve TME remodeling and immune response activation. In summary, the construction and delivery strategy of RUT-RPP-LP may provide new ideas and approaches for enhancing tumor immunotherapy efficacy and addressing the challenge of insufficient tumor immunogenicity.

## 2. Materials

### 2.1. Reagents

Methanol and methylene chloride were sourced from Tianjin Fuyu Fine Chemicals Co., Ltd. (Tianjin, China). Chloroform, 5-amino-1-pentanol, and 1,4-butanediol diacrylate were acquired from Aladdin Reagents Co., Ltd. (Shanghai, China). Polyethyleneimine (PEI), dimethyl sulfoxide, RES, and RUT were purchased from Merck Chemical Co., Ltd. (Darmstadt, Germany). Dioleoyl phosphatidylethanolamine (DOPE) and cholesteryl hemisuccinate (CHEMS) were obtained from Shanghai Macklin Biochemical Technology Co., Ltd. (Shanghai, China). Phosphotungstic acid staining solution was supplied by Zhongmirror Science Instrument Technology Co., Ltd. (Beijing, China). Penicillin-streptomycin solution double antibody and phosphate-buffered solution (PBS) were acquired from Dalian Meilun Biotechnology Co., Ltd. (Dalian, China). Fetal bovine serum (FBS) was purchased from Ecosas Biotech Co., Ltd. (Suzhou, China). Trypsin cell digest, CCK-8 kit, neutral tree glue, hematoxylin staining solution, eosin staining solution, Ki-67 antibody, immunostaining permeability solution, Triton X-100, 4′,6-diamidino-2-phenylindole (DAPI), and anti-fluorescence quenching tablet sealant were sourced from Biyuntian Biotechnology Co., Ltd. (Shanghai, China). 1,1-dioctadecyl-3,3,3,3-tetramethylindotricarbocyanine iodide (DiR) fluorescent dye was obtained from Shanghai Aladdin Biochemical Technology Co., Ltd. (Shanghai, China). Hematoxylin and eosin stain (H&E) solution kit, ethylene diamine tetraacetic acid (EDTA) antigen repair solution, and 3,3′-diaminobenzidine (DAB) chromogener of histochemical kit were supplied from Wuhan Sevier Biotechnology Co., Ltd. (Wuhan, China). Absolute ethanol and xylene were acquired from Sinopdrug Group Chemical Reagent Co., Ltd. (Shanghai, China). The 4% paraformaldehyde was purchased from Solebo Biotech Co., Ltd. (Beijing, China). Terminal deoxynucleotidyl transferase dUTP nick end labeling (TUNEL) kit and Enzyme-linked immunosorbent assay kit (ELISA) were obtained from Shanghai Enzyme Immunoassay Biotechnology Co., Ltd. (Shanghai, China). PD-L1 antibody, CRT antibody, and HMGB1 antibody were sourced from Wuhan Sanying Biotechnology Co., Ltd. (Wuhan, China). RPMI-1640 medium was supplied from Thermo Fisher Scientific Inc. (Waltham, MA, USA).

### 2.2. Cell Lines and Animals

Mouse embryonic fibroblast 3T3 (Cat. No. CC-Y2016) and mouse colorectal cancer cells CT26 (Cat. No. CC-Y2158) were purchased from Shanghai Enzyme Research Biotechnology Co., Ltd. (Shanghai, China). All cells were cultured in RPMI-1640 medium (Thermo Fisher Scientific Inc., Waltham, MA, USA) with 10% FBS at 37 °C under a humidified atmosphere containing 5% CO_2_.

Female Balb/c mice (aged 6–8 weeks, 20 ± 2 g) were supplied from Liaoning Changsheng Animal Center (Benxi, China). All animal procedures were performed in accordance with the protocol approved by the Animal Experiment Ethics Committee of Heilongjiang University of Traditional Chinese Medicine (No. 2024121004).

## 3. Method

### 3.1. Preparation of RPP

Poly (β-amino ester) (PBAE) and Polyethyleneimine-poly (β-amino ester) (PP) were synthesized via Michael addition reaction, and RPP was prepared by self-assembly and organic solvent volatilization [17]. 5-amino-1-pentanol (130.1 mg) and 1,4-butanediol diacrylate (333.33 mg) were dissolved completely in dichloromethane (10 mL). The reaction mixture was magnetically stirred in an oil bath at 65 °C for 72 h. Then, PBAE was isolated by extraction with diethyl ether followed by vacuum drying. PBAE (461.85 mg) and PEI (90 mg) were precisely weighed, dissolved in DMSO (14 mL), and stirred at 25 °C and 1000 rpm for 24 h. Subsequently, the solution was transferred to a dialysis bag (3.5 kDa) for continued dialysis for 24 h, and the double-distilled water was changed four times. The solution completed by dialysis was then freeze-dried to afford PP. Finally, the PP copolymer and RES (mass ratio 5:1) dissolved in methanol were used as the organic phase (2 mL in total), and 50 mL of the aqueous phase (pH 7.4, 6 s·drop^−1^) was slowly injected under magnetic stirring at 25 °C and 1000 rpm. After complete evaporation of methanol, RPP was obtained via filtration through a 0.22 μm filter membrane.

### 3.2. Preparation of RUT-RPP-LP

RUT-RPP-LP was prepared via thin film dispersion. DOPE and CHEMS (4:1, *w*/*w*) were dissolved in an equal volume of chloroform to form the lipid phase. RUT and RPP (2:1, *w*/*w*) were dissolved in methanol and PBS, respectively, and the drug–lipid ratio was maintained at 1:10 (the PBS of RPP was used as the hydration solution). The lipid phase and RUT methanol solution were then transferred to an eggplant-shaped flask. A rotary evaporator was used at 40 °C and 50 rpm for 2 h to form a uniform thin film on the inner wall of the flask. The hydration solution was added to the flask and hydrated (50 °C, 80 r·min^−1^) until the film completely detached. After cooling the solution to 25 °C, RUT-RPP-LP was obtained by ultrasonic crushing in an ice bath for 20 min, followed by filtration through a 0.45 μm filter membrane to remove impurities. The preparation process and expected structure of RUT-RPP-LP are described in Scheme 1a. DiR@RUT-RPP-LP, Blank-LP, PP-LP, RUT-LP, and RPP-LP were prepared via the identical procedure, with only the composition of the lipid phase or hydration solution varied accordingly.

### 3.3. HPLC Method

The contents of RUT and RES were quantified using an HPLC (LC-20AT, Shimadzu Co. Ltd., Kyoto, Japan). Chromatographic separation was achieved on a DIKMA C18 column (250 mm × 4.6 mm, 5 µm) at 25 °C. The mobile phase consisted of 0.1% glacial acetic acid and methanol (45–50%) with gradient elution for 0–20 min at a flow rate of 1 mL·min^−1^. A 10 μL sample was injected, and RUT and RES were detected at 257 and 306 nm wavelengths, respectively. Therein, the calibration curve equation of RUT is y = 18698x − 22932 (within the concentration range of 6.00 to 240.00 μg·mL^−1^), corresponding to the limit of quantification (LOQ) and detection (LOD) being 1.86 μg·mL^−1^ and 5.64 μg·mL^−1^, respectively. The calibration curve equation of RES is y = 71080x + 45037 (within the concentration range of 5.00 to 200.00 μg·mL^−1^), with LOQ and LOD being 1.55 μg·mL^−1^ and 4.70 μg·mL^−1^, respectively.

### 3.4. Characterization of RUT-RPP-LP

#### 3.4.1. Morphology

The RUT-RPP-LP was dropped onto the carbon-coated copper grid and left standing for 10 min at 25 °C under ambient dark conditions. After drying, 2% phosphotungstic acid negative staining solution was added for 30 s. Next, excess staining solution was removed via suction with filter paper and stored in a desiccator in the dark for 12 h. Finally, the fully dried specimen was imaged using a transmission electron microscope (TEM, JEM-F200, Japan Electronics Co. Ltd., Tokyo, Japan).

#### 3.4.2. Particle Size, Polydispersity Index (PDI) and Zeta Potential

Equal volumes of blank liposomes, RUT-LP and RUT-RPP-LP were individually diluted to appropriate concentrations and added to a quartz cell. The particle size, PDI, and zeta potential of the samples were determined via dynamic light scattering (DLS) using a Malvern particle size analyzer (Masterizer 2000, Malvern Instruments Ltd., Malvern, UK). The particle size, PDI, and zeta potential of RPP were measured using the same procedure [17].

#### 3.4.3. Storage Stability

The leakage rate (LR) refers to the change in encapsulation efficiency of liposomes during storage and reflects the physical stability of liposomes [21]. The RUT-RPP-LP were stored at 4 °C in the dark. Particle size and PDI of RUT-RPP-LP were determined at 0, 1, 3, 7, 10, and 15 days using DLS. Meanwhile, the drug content was determined via HPLC to calculate the encapsulation efficiency (EE) at each time point, and the LR was calculated according to the following formula:

(1) $$\mathrm{EE}\left(\%\right)=\;\left[\frac{\left(W_{2}-W_{1}\right)}{W_{2}-W_{1}+W_{3}}\right]\;\times \;100\%$$

(2) $$\mathrm{LR}\left(\%\right)=\left(1-\frac{{\mathrm{EE}}_{\mathrm{after}\mathrm{storage}}}{{\mathrm{EE}}_{\mathrm{before}\mathrm{storage}}}\right)\;\times \;100\%\;$$

where W_1_ was the amount of unencapsulated free drug, W_2_ was the amount of the total drug, W_3_ was the mass of the blank vector PP-LP, EE_after storage_ was the EE of RUT-RPP-LP after storage for a certain time, and EE_before storage_ was the EE of RUT-RPP-LP before storage, respectively.

### 3.5. In Vitro Studies of pH-Responsive Drug Release

The RUT-RPP-LP was added to 100 mL phosphate buffer (0.01 mol·L^−1^) at pH 5.5, 6.8, and 7.4, respectively. Then, each dispersion was incubated in a constant-temperature shaking incubator at 37 °C and 100 rpm. The samples were taken at 0, 4, 8, 12, 16, 20, and 24 h. The particle size of the samples was measured via DLS, and the response curve of particle size with pH value and time was plotted.

Another indicator of the pH response of RUT-RPP-LP is the release rate, which is the degree of drug release stimulated by different pH conditions that mimic the physiological environment. To systematically evaluate the pH-dependent in vitro drug release profile of RUT-RPP-LP, dialysis bags (10 kDa) containing 2 mL of RUT-RPP-LP were immersed in 100 mL of PBS individually adjusted to pH 5.5, 6.8, or 7.4 and continuously shaken in a constant temperature shaker (KYC 100B, Shanghai FUMA Experimental Equipment Co., Ltd., Shanghai, China) at 37 °C and 100 rpm. At 1, 2, 4, 6, 8, 12, 24, 48, and 72 h, 1 mL of release solution was withdrawn and immediately replaced with an equal volume of pre-warmed fresh medium. Two parallel groups were established. Next, the RUT and RES contents in the samples were determined via HPLC, and the cumulative drug release rate (Q_i_) was calculated according to the following formula:

(3) $$Q_{i}\left(\%\right)\;=\;\left[\frac{V_{1}\;\times \;\left(C_{1}\;+\;C_{2}\;+\;\ldots \;+\;C_{i\;-\;1}\right)\;+{\;V}_{2}\;\times \;C_{i}}{W}\right]\;\times \;100\%\;$$

where V_1_ was the volume of each sampling and V_2_ was the volume of medium. C_1_, …, C_i_ was the concentrations of RUT/RES at each time point, and W was the total amount of drug in RUT-RPP-LP.

### 3.6. CCK-8 Assay

Cell suspensions of 3T3 and CT26 cells in the logarithmic growth phase were prepared in complete medium and seeded into 96-well plates at 6 × 10^3^ cells·well^−1^. Following overnight cultivation, 100 μL of various concentrations of PP-LP (0, 200, 400, 600, 800, and 1000 μg·mL^−1^), RUT-LP, RPP-LP, free RUT + RES, and RUT-RPP-LP were added. The concentrations of RUT and RES were 0, 5, 10, 20, 40, 80 μg·mL^−1^ and 0, 2.5, 5, 10, 20, 40 μg·mL^−1^, respectively. In addition to the blank control group and the negative control group, six parallel wells were set up in each group. After continued incubation for 24 h, CCK-8 reagent (10 µL) was added to each well and cultured for 1 h. The OD at 450 nm was measured using a microplate reader (SH-1, BioTek Instruments, Inc., Winooski, VT, USA). The cell viability was calculated according to the following formula, and the half maximal inhibitory concentration (IC_50_) was calculated via GraphPad Prism 9.5:

(4) $$\mathrm{Cell}\;\mathrm{viability}\left(\%\right)\;=\;\left[\frac{{\mathrm{OD}}_{\mathrm{treat}}-{\mathrm{OD}}_{\mathrm{blank}}}{{\mathrm{OD}}_{\mathrm{negative}\;\mathrm{control}}-{\mathrm{OD}}_{\mathrm{blank}}}\right]\;\times \;100\%\;$$

where OD_treat_ was the OD value of each experimental group, OD_blank_ was the OD value of the blank control group, and OD_negative control_ was the OD value of the negative control group, respectively.

### 3.7. Mice Syngeneic and Drug Administration

A cell suspension of 1 × 10^7^ cells·mL^−1^ was obtained via trypsin digestion of CT26 cells in logarithmic growth phase, followed by centrifugation, resuspension in sterile PBS, and cell counting. The density was empirically established in preliminary studies. Following a week of acclimatization in a specific pathogen-free facility, syngeneic mouse models were established via subcutaneous injection of 100 μL of the cell suspension into the right flank of Balb/c mice. When the primary tumor volume reached 100 mm^3^, tumor-bearing mice were randomly divided into five groups (n = 5): control group (normal saline), free RUT + RES group, RUT-LP group, RPP-LP group, and RUT-RPP-LP group. All groups were administered intravenously via tail vein injection at equal doses corresponding to RUT (4 mg·kg^−1^) and RES (2 mg·kg^−1^) every two days for six consecutive administrations [22,23].

### 3.8. Biodistribution Imaging

DiR, a lipophilic near-infrared carbocyanine dye (Ex/Em = 740/780 nm), stably incorporates into lipid bilayers and enables deep-tumor imaging [24]. Accordingly, DiR was selected as the fluorescence signal source. Two groups of randomly assigned tumor-bearing mice were injected with free DiR solution or DiR@RUT-RPP-LP via the tail vein, respectively. The dose of DiR was 200 μg·kg^−1^. Mice were sacrificed at 1, 4, 12, and 24 h after administration, and their heart, liver, spleen, lung, kidney, and tumors were removed, washed with normal saline, and biodistribution was monitored using a small animal in vivo imaging system (Maestro EX, Photometrics, Tucson, AZ, USA). Meanwhile, the fluorescence intensity of each organ was recorded, and semi-quantitative analysis of organ fluorescence was performed.

### 3.9. In Vivo Anti-Tumor Studies

#### 3.9.1. Anti-Tumor Effect Study

Throughout the treatment period, body weights of tumor-bearing mice in each group (n = 5) were monitored regularly, the tumor diameter was measured using a vernier caliper, and the tumor volume was calculated according to the following formula:

(5) $$V_{\mathrm{tumor}}\left({\mathrm{mm}}^{3}\right)\;=\;\frac{L\;\times {\;W}^{2}}{2}\;$$

where L was the length of the tumor, and W was the width of the tumor.

On day 20 post-treatment initiation, mice were humanely euthanized. Meanwhile, the main organs (heart, liver, spleen, lung, and kidney) and tumors were isolated, washed with normal saline, and dried with filter paper. Tumors from each group were individually weighed, and the tumor growth inhibition rate (TIR) was computed using the following formula:

(6) $$TIR\left(\%\right)\;=\;\left(\frac{m_{1}-m_{2}}{m_{1}}\right)\;\times \;100\%\;$$

where m_1_ and m_2_ were the tumor weights of the control and treated groups, respectively.

#### 3.9.2. Treatment Quality of Life Assessment

Survival was recorded by counting the days from day 1, when tumor-bearing mice (n = 10) were administered the drug, until day 60 [25]. Simultaneously, tumor-bearing mice were monitored daily for activity and mortality. Ultimately, the median survival time was estimated via the Kaplan–Meier method, and the survival curve was plotted with GraphPad Prism 9.5.

### 3.10. Histopathology Analysis

Tumor tissues and major organs were processed for histopathological analysis following the established procedures [26]. The tumor-bearing mice were humanely euthanized and dissected. Then, tumor, liver, kidney, spleen, heart, and lung tissues were collected and fixed in 4% paraformaldehyde. The organs were embedded in paraffin blocks and cut into 5 μm sections. Sections were deparaffinized in xylene, then hydrated in progressively decreasing ethanol concentrations (100%, 95%, 90%, 80%, and 70%), rinsed in distilled water, and stained with H&E. Stained sections were dehydrated in a graded ethanol series (70%, 80%, 90%, 95%, and 100%) and cleared in xylene. Ultimately, sections were sealed using neutral gum and observed under a light microscope (Eclipse E100, Nikon Corporation, Tokyo, Japan). Tumor tissue sections prepared via identical protocols were additionally used for Ki-67 staining, TUNEL staining, and immunohistochemistry.

### 3.11. Ki-67 Staining and TUNEL Staining

Following antigen retrieval, tumor tissue sections were blocked with 3% BSA at 25 °C for 1 h. After discarding the blocking solution, the sections were incubated with primary anti-Ki-67 antibody at 4 °C overnight, rinsed with PBS, and then cultivated with fluorescent secondary antibody at 25 °C for 1 h. After PBS washing, the sections had DAPI added dropwise and were cultured for 10 min. Following another wash, the sections were treated with an anti-fluorescence quenching agent, mounted on a slide, and visualized via fluorescence microscope (CFM-500, Olympus Corporation, Tokyo, Japan). The cell proliferation index (PI) was quantified using ImageJ 1.54f software.

Tumor cell apoptosis was further assessed via TUNEL staining. Tumor tissue sections were incubated in PBS containing 0.1% Triton X-100 for 20 min, and then continued to be cultured in PBS containing 20 µg·mL^−1^ proteinase K for another 20 min. The sections were washed three times with PBS to completely remove the proteinase. Meanwhile, the TUNEL reaction mixture was prepared, added to the sections, and cultivated in the dark for 1 h. These operations were carried out at 37 °C. After three washes, DAPI was added to the sections and incubated in the dark at 25 °C for 10 min. The sections were then washed, mounted using anti-fluorescence quenching medium, and observed under a fluorescence microscope. Additionally, the ImageJ software was employed to quantify the apoptosis index (AI).

### 3.12. Immunohistochemistry (IHC)

The tissue sections were heated in the repair box for 10 min, incubated with 3% H_2_O_2_ in the dark for 20 min, and washed three times with PBS. Nonspecific binding sites were blocked using 3% bovine serum albumin. Primary antibodies targeting PD-L1, CRT, and HMGB1 were applied overnight at 4 °C. Subsequently, incubation with species-appropriate secondary antibodies for 1 h at 25 °C under light-protected conditions. Immunoreactivity was visualized via DAB until a distinct brown-yellow precipitate formed, and then terminated with distilled water. Later, the sections were counterstained with hematoxylin for 2 min, rinsed, differentiated for 30 s, and blued for 1 min. Finally, the sections were dehydrated, cleared, mounted, and imaged under a light microscope.

### 3.13. Analysis of Cytokine Levels

Tumor tissues from each group were minced, weighed, and then homogenized in ice-cold PBS (1:5, *w*/*v*) availing high-speed tissue grinder (KZ-II, Shanghai Peiaim Optical Instrument Manufacturing Co., Ltd., Shanghai, China). Following centrifugation at 3000 rpm for 10 min, supernatants were collected. Levels of tumor necrosis factor-α (TNF-α) and Interferon-γ (IFN-γ) in tumor tissue homogenates were examined via ELISA according to the manufacturer’s guidelines.

### 3.14. Statistical Analyses

Measurements were conducted in triplicate, and results were expressed as mean ± standard deviation. Data analysis was performed via GraphPad Prism, ImageJ, and Origin 2021. Quantitative evaluation employed a one-way analysis of variance (ANOVA) or the Student’s t-test unless specified otherwise. Additionally, * *p* < 0.05, ** *p* < 0.01 and *** *p* < 0.001 were statistically significant.

## 4. Results and Discussion

### 4.1. Characterization of RUT-RPP-LP

#### 4.1.1. Morphology and DLS Analysis

**Figure 1:**
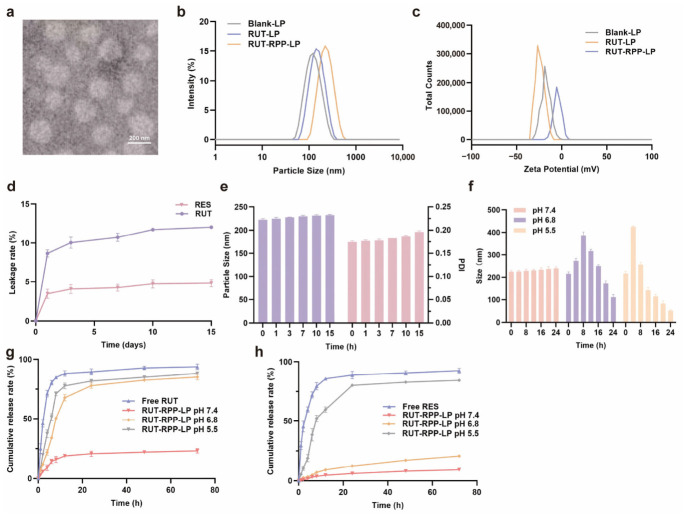
Characterization of RUT-RPP-LP. TEM of RUT-RPP-LP (**a**). Particle size (**b**) and zeta potential (**c**) of Blank-LP, RUT-LP, and RUT-RPP-LP. Changes in LR (**d**), particle size, and PDI (**e**) of RUT-RPP-LP. pH Sensitivity of RUT-RPP-LP (**f**). In vitro pH release of RUT (**g**) and RES (**h**).

Under TEM observation (Figure 1a), RUT-RPP-LP showed a spherical shape, good dispersion, uniform particle size, and excellent monodispersity. Meanwhile, blank liposomes exhibited an average particle size of 121.81 ± 2.06 nm and a PDI of 0.086 ± 0.008, as shown in Figure 1b. Upon incorporation of RUT into the lipid bilayer, the average particle size and PDI of RUT-LP increased to 139.14 ± 1.79 nm and 0.166 ± 0.027, respectively. Subsequent loading of RPP into the liposomal core further increased the hydrodynamic diameter to 218.88 ± 2.20 nm (<300 nm) with a PDI of 0.187 ± 0.033 (<0.300). The RUT-RPP-LP meets the requirements of passive targeting of solid tumor tissue EPR effect, which could achieve efficient penetration and long-term retention of tumor tissue to enhance the targeted accumulation efficiency [27].

At neutral pH, DOPE remains overall electrically neutral, whereas CHEMS, as acidic lipids, had complete deprotonation of their carboxylic acid groups, thereby conferring a pronounced negative charge to blank liposomes [28]. Therefore, the zeta potentials of Blank-LP, RUT-LP and RUT-RPP-LP were −20.92 ± 0.63, −24.37 ± 0.49 and −4.56 ± 0.21 mV (Figure 1c). The potential reduction in RUT-LP may be related to the enhanced surface-negative charge density arising from exposure of phenolic hydroxyl groups upon incorporation of RUT into the lipid bilayer [29]. Additionally, the marked increase for RUT-RPP-LP reflected electrostatic neutralization between the positively charged RPP (the particle size, PDI, and zeta potential were 83.81 nm, 0.22, and 14.78 mV, respectively) and the negatively charged liposomal membrane [17]. Specifically, cationic RPP were encapsulated within the anionic liposomal lumen, forming a charge-balanced LMH via strong electrostatic interactions [17].

#### 4.1.2. Storage Stability

Within 15 days of storage at 4 °C in the dark, the cumulative leakage of RUT in RUT-RPP-LP remained below 12%, whereas that of RES was less than 3% (Figure 1d,e). The comparatively higher leakage of RUT was likely attributable to its localization within the lipid bilayer, which is more susceptible to fluctuations in membrane fluidity. In contrast, RES was encapsulated in the core of RPP, where it benefits from a stronger physical barrier, thereby exhibiting superior retention. Furthermore, the particle size and PDI of RUT-RPP-LP changed minimally within 15 days, indicating the preservation of lipid membrane integrity, structural stability of the hybrid formulation, and sustained high-efficiency drug encapsulation.

### 4.2. In Vitro pH Sensitivity Evaluation

The structural stability of RUT-RPP-LP and its pH-responsive behavior under acidic conditions were evaluated in Figure 1f. Under physiological conditions (pH 7.4), the particle size fluctuated less than 20 nm, indicating preserved integrity of the lipid bilayer. At this pH, DOPE is electrically neutral, whereas CHEMS carries a negative charge owing to carboxyl group deprotonation [30]. This complementary charge interaction between DOPE and CHEMS stabilizes the DOPE lamellar phase and promotes bilayer formation, thereby preserving the structural integrity of RUT-RPP-LP. When exposed to a TME micro-acid environment (pH 6.8), partial protonation of CHEMS carboxyl groups reduced the surface negative charge, triggering transient nanoparticle aggregation (evidenced by an initial size increase), followed by DOPE-mediated lamellar-to-hexagonal phase transition [31]. Sequentially, RUT and RPP were released. Under strongly acidic lysosomal conditions (pH 5.5), the response process was significantly accelerated (peaking at 4 h), and the drastic conformational rearrangement of CHEMS induced rapid lipid membrane destabilization, which simultaneously triggered the dissociation of the core micelle (PBAE/PEI amino protonation). After 16 h, the particle size declined sharply to below 100 nm, enabling hierarchical release of RUT and RES. Collectively, these findings demonstrated that RUT-RPP-LP exhibited dual pH response characteristics of the TME and lysosomal acidic environment and achieved spatiotemporally controlled, cascade-triggered dual-drug release via preferential liposomal disintegration and subsequent micellar dissociation.

### 4.3. In Vitro pH Release Study

The dynamic release of RUT and RES was investigated using the dynamic dialysis method, with results presented in Figure 1g,h. Under physiological neutral conditions (pH 7.4), the cumulative release of RUT and RES was merely 23% and 8% over 72 h, which was substantially lower than that of the free drug (more than 90% under identical conditions). This was attributed to the structural integrity and high encapsulation stability conferred by the compact lipid bilayer, which resulted from the charge-mediated synergistic interaction between DOPE and CHEMS. In the condition of TME (pH 6.8), 80% of RUT was released rapidly within 24 h due to DOPE phase transition-driven membrane disintegration, while RES was still stable (less than 20% within 72 h), owing to the pH buffering ability of the PP copolymer in the RPP. At pH 5.5, complete protonation of the amine groups in the PBAE block disrupted the hydrophobic–hydrophilic balance, triggering micelle core dissociation and the consequent burst release of RES, with 80% cumulative release within 24 h.

The in vitro pH release profile aligned closely with the particle size change observed in the pH sensitivity investigation, corroborating the direct correlation between RUT release and dynamic changes in membrane structure. The behavior further confirmed the intelligent release mechanism of RUT-RPP-LP. Specifically, structural homeostasis was maintained via molecular complementation in the neutral environment to ensure that the drug remains encapsulated during circulation. Under acidic TME and lysosome conditions, structural destabilization occurred rapidly through the charge state, triggering membrane structural change and dissociation.

### 4.4. Results of CCK-8 Assay

**Figure 2:**
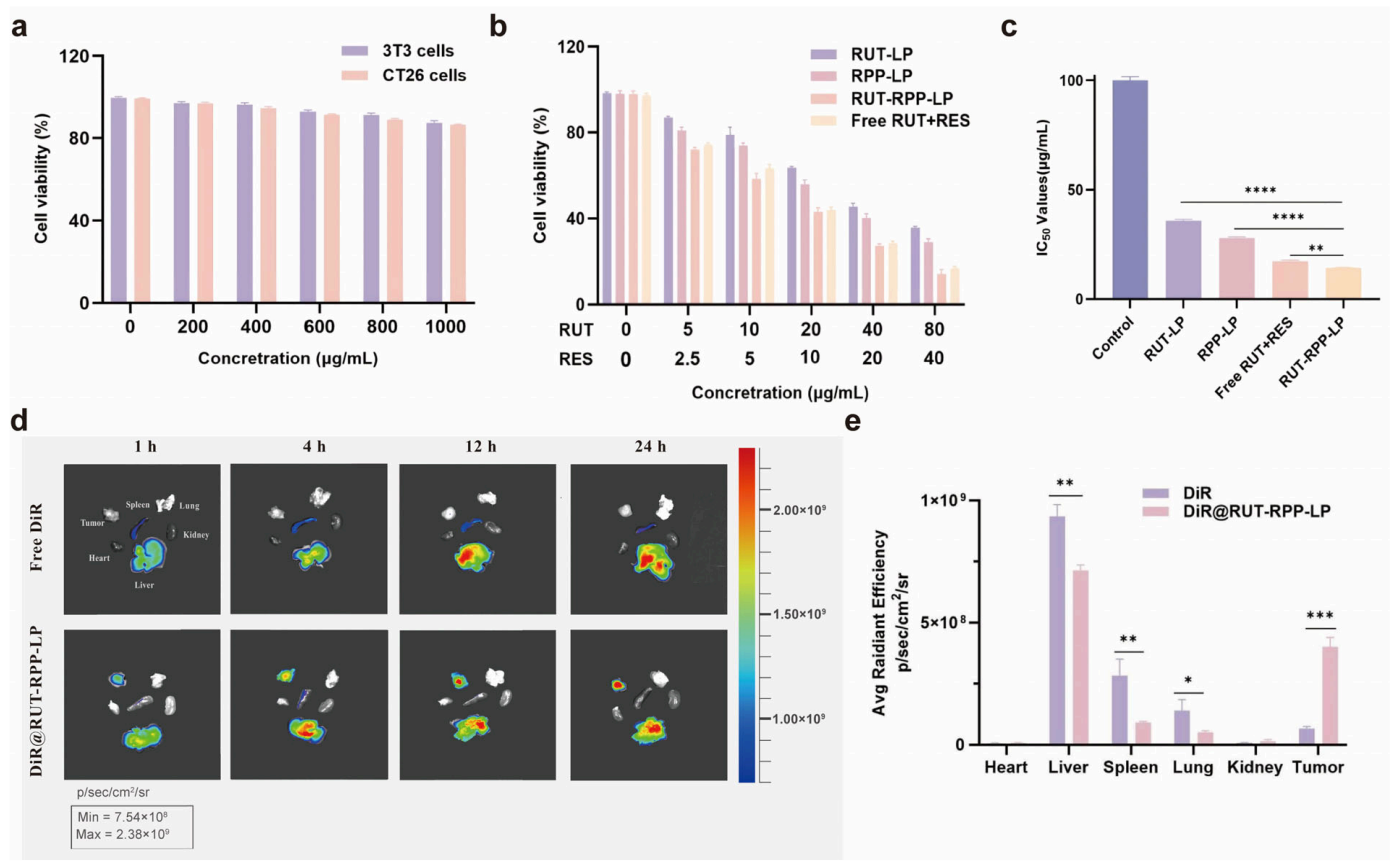
The cytotoxicity and in vivo targeting properties of RUT-RPP-LP. Effects of different concentrations of blank carriers on the activity of 3T3 and CT26 cells (**a**). Cell viability of CT26 cells under different treatment groups and administration concentrations (**b**), and IC_50_ values of CT26 in different treatment groups (**c**). Fluorescence distribution of organs in vivo for RUT-RPP-LP (**d**). 24 h organ fluorescence semi-quantification (**e**). * *p* < 0.05, ** *p* < 0.01, *** *p* < 0.001 and **** *p* < 0.0001 were statistically significant.

As the drug concentration increased, the cell viability decreased slightly, but the overall reduction was negligible. Even at the maximum concentration, the cell viability remained above 85%, as illustrated in Figure 2a. These results proved that RUT-RPP-LP demonstrated favorable biocompatibility and was suitable for in vivo delivery applications.

The inhibitory effects of free RUT + RES, RUT-LP, RPP-LP, and RUT-RPP-LP on CT26 cell proliferation were systematically evaluated using the CCK-8 assay and IC_50_ values (Figure 2b,c). The cell viability of each group decreased in a dose-dependent manner. Notably, the RUT-RPP-LP group was reduced most pronouncedly, indicating superior antiproliferative activity. Consistently, RUT-RPP-LP displayed the lowest IC_50_ value, which was significantly lower than those of free RUT + RES, RUT-LP, and RPP-LP. RUT-RPP-LP achieved co-encapsulation of both drugs via LMH, which not only markedly improved the drug solubility and stability, but also incorporated pH-responsive carrier components to facilitate spatiotemporally controlled, stepwise drug release under acidic microenvironments. These attributes exhibited important prospects for RUT-RPP-LP in overcoming the limitations of monotherapy and enhancing synergistic effects.

### 4.5. In Vivo Targeted Delivery and Retention of RUT-RPP-LP

The fluorescence distribution and semi-quantitative analysis of isolated mouse organs and tumor tissues at multiple time points were presented in Figure 2d,e. In the free DiR group, the fluorescence signal was mainly localized in organs with a rich reticuloendothelial system, such as the spleen and liver, and only minimal accumulation was detected in tumor tissues. However, the DiR@RUT-RPP-LP group exhibited pronounced fluorescence accumulation at the tumor site 1 h after tail vein injection, and the signal intensified progressively over time, demonstrating a significant time-dependent cumulative effect. Moreover, the fluorescence intensity of the DiR@RUT-RPP-LP group in normal organs was significantly lower than that of the free DiR group, while tumor-specific fluorescence intensity was markedly higher than that of the control group, indicating RUT-RPP-LP could prolong the in vivo circulation and enhance the passive targeting of tumors through the EPR effect.

The abnormal structure of tumor blood vessels and the dysfunction of lymphatic excretion notably promoted the penetration and retention of nanoparticles in the tumor site. In contrast, healthy tissues exhibited intact vascular endothelial barriers and efficient systemic clearance mechanisms, thereby restricting nonspecific nanoparticle accumulation [32]. Thus, DiR@RUT-RPP-LP leveraged the pathophysiology-driven selectivity to significantly enhance drug enrichment at the tumor site, concurrently mitigating off-target exposure and associated systemic toxicity. The dual benefit underscored the potential advantages of DiR@RUT-RPP-LP in clinical applications.

### 4.6. In Vivo Therapeutic Efficacy of RUT-RPP-LP

#### 4.6.1. Study on the Anti-Tumor Effect

**Figure 3:**
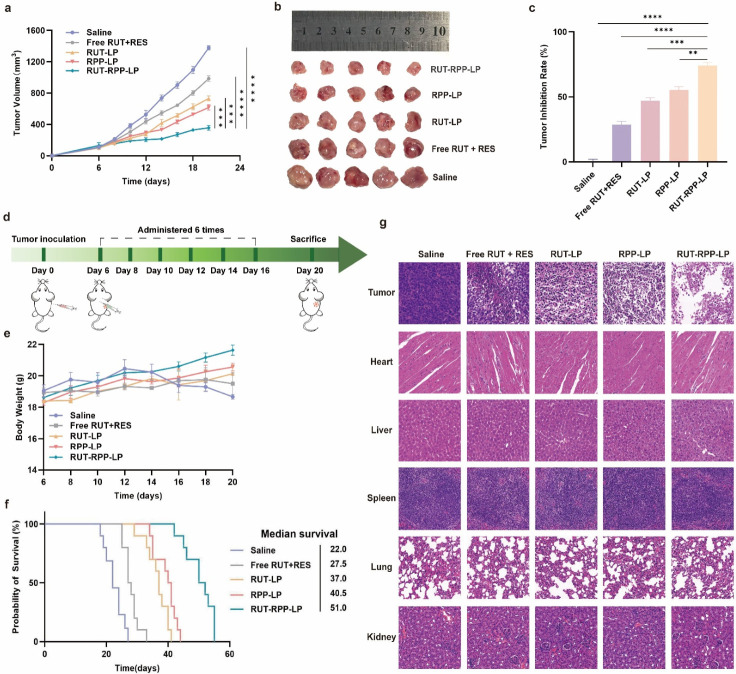
In vivo anti-tumor treatment study of RUT-RPP-LP. Tumor volume change curve, n = 5 (**a**). In vitro tumor efficacy diagram, n = 5 (**b**). Tumor inhibition rate, n = 5 (**c**). Treatment scheme for Balb/c-bearing mouse (**d**). Weight change in tumor-bearing mice, n = 5 (**e**). Survival curve and median survival time of tumor-bearing mice, n = 10 (**f**). H&E staining results of tumor tissue and major organs, and the scale bar was 50 μm (**g**). ** *p* < 0.01, *** *p* < 0.001 and **** *p* < 0.0001 were statistically significant.

Balb/c tumor-bearing mice were treated according to the administration regimen (Figure 3d). Based on the conventional dose range of RUT and RES and the process optimization of drug-loading ratio (2:1, *w*/*w*) in RUT-RPP-LP, the administration concentration was set as RUT 4 mg·kg^−1^ and RES 2 mg·kg^−1^ [22,23,32]. As shown in Figure 3a, tumor volume in the saline group increased rapidly following tumor inoculation, thereby confirming successful establishment of the syngeneic tumor model. Among all groups, the saline group displayed the steepest tumor growth slope, whereas the RUT-RPP-LP group exhibited the slowest tumor progression. Final tumor volumes measured on day 20 were depicted in Figure 3b, and corresponding TIR was calculated and summarized in Figure 3c. The ascending order of the TIR values was the free RUT + RES group, the RPP-LP group, the RUT-LP group, and the RUT-RPP-LP group. The above results were consistent with the in vitro cytotoxicity experiments, thereby affirming the therapeutic superiority of RUT-RPP-LP.

Although the free RUT + RES group modestly suppressed tumor growth, the free drug mainly relies on the blood circulation to passively diffuse into the TME and lacks the specific targeting ability to tumor tissue, resulting in low accumulation of drug in the tumor site and limited anti-tumor effect. In contrast, RUT-RPP-LP achieved selective and enriched accumulation within tumor tissue via the pathological hyperpermeability of tumor vasculature, showing markedly superior tumor growth inhibition. Moreover, RUT-RPP-LP exhibited stronger anti-tumor efficacy than RUT-LP and RPP-LP, underscoring the synergistic advantage conferred via RUT-RPP-LP in enhancing in vivo anti-tumor activity through the co-delivery of RUT and RES.

#### 4.6.2. Quality of Life of Tumor-Bearing Mice After Treatment

The weight of the mice generally increased throughout the experimental period in all treatment groups (Figure 3e), without observable signs of systemic toxicity (loss of flesh, abnormal mental status, or other adverse clinical manifestations), supporting the favorable biosafety of each agent. Nevertheless, mice in the saline group displayed progressive body weight loss, likely attributable to tumor-induced metabolic stress and impaired nutrient absorption secondary to extensive tumor burden. Notably, Kaplan–Meier survival analysis (Figure 3f) revealed a significant extension of the survival time in the RUT-RPP-LP group (significantly better than that of the RUT-LP group and RPP-LP group), corroborating that RUT-RPP-LP substantially improved both therapeutic efficacy and treatment tolerability. Furthermore, mice treated with RUT-RPP-LP exhibited markedly preserved quality of life, including normal activity and stable coat condition, indicating superior treatment safety. It can be seen that the RUT-RPP-LP utilized the LMH structure for the co-delivery of RUT and RES, and may achieve a synergistic therapeutic effect without compromising biological safety.

#### 4.6.3. Results of H&E Staining

Histopathological examination of tissues and organs was performed using H&E staining, with representative images presented in Figure 3g. In the saline group, the tumor tissue structure was complete, the cells were closely distributed, the nucleus was deep blue, the cytoplasm was red, and the morphologies of both were intact, with no evidence of structural damage. However, all groups displayed varying degrees of tumor necrosis, characterized by cellular disarray, nuclear pyknosis, and cytoplasmic dissolution. Notably, the RUT-RPP-LP group showed the most extensive necrotic areas marked by sharply demarcated zones of coagulative necrosis, severe disruption of cellular architecture, and pronounced loss of cellular integrity. The findings indicated that RUT-RPP-LP exerted potent anti-tumor activity, significantly enhancing tumor cell cytotoxicity and suppressing tumor cell invasion.

H&E staining was further performed on the major organs (heart, liver, spleen, lung, and kidney) of tumor-bearing mice (Figure 3g). The organ histological structure of mice in each group remained intact, and no obvious pathological damage was observed, collectively supporting the favorable in vivo biosafety of RUT-RPP-LP.

### 4.7. The Synergistic Effect of RUT-RPP-LP

#### 4.7.1. Tumor Cell Proliferation and Apoptosis

**Figure 4:**
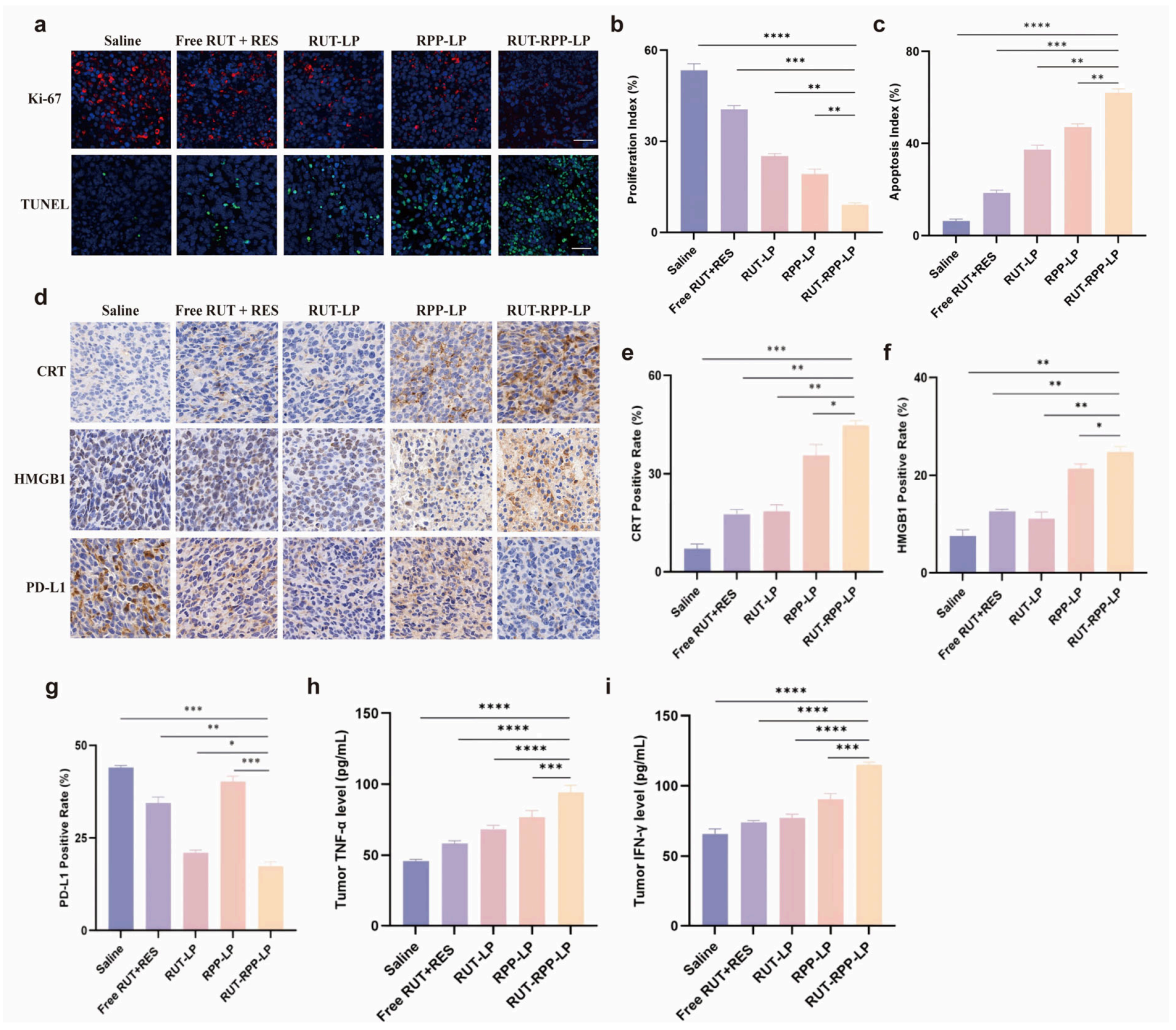
In vivo anti-tumor mechanism of RUT-RPP-LP. Ki-67 and TUNEL fluorescence staining of tumor tissues in different treatment groups, and the scale bar was 50 μm (**a**). Cell PI of different treatment groups (**b**). AI of cells in different treatment groups (**c**). Protein expression in tumor tissue of different treatment groups, and the scale bar was 50 μm (**d**). The positive cell rates of CRT (**e**), HMGB1 (**f**), and PD-LI (**g**) proteins in different treatment groups. Content of TNF-α (**h**) and IFN-γ (**i**) in tumor tissue, n = 6. * *p* < 0.05, ** *p* < 0.01, *** *p* < 0.001 and **** *p* < 0.0001 were statistically significant.

The proliferation status of tumor cells was reflected via the fluorescence expression level after Ki-67 staining, and the PI was calculated, as presented in Figure 4a,b. The red fluorescence in the saline group was the most obvious, with a PI of 52.74 ± 2.16%, consistent with robust tumor cell proliferation. Nevertheless, the free RUT + RES group showed a decreased PI of 40.47 ± 1.28%, indicating moderate antiproliferative activity. Both RUT-LP and RPP-LP significantly lowered the PI, demonstrating that the EPR effect could also be achieved through the single-drug nanodrug delivery system. Apparently, the RUT-RPP-LP group displayed the strongest suppression of proliferation, with diminishing Ki-67 staining and yielding the lowest PI (9.11 ± 0.68%). The results indicated that the combination of RUT and RES significantly inhibited the proliferation of tumor cells.

TUNEL staining (Figure 4a) was performed to assess apoptosis of tumor cells in each group, and green fluorescence represented apoptotic cells [33]. AI was quantified and presented in Figure 4c. In the normal saline control group, only faint green fluorescence was observed, with a mean AI of 6.36 ± 0.82%, indicating minimal baseline apoptosis. However, the increased AI of the free RUT + RES group suggested initial activation of the apoptotic pathway by the drug combination. The apoptotic cells in the RUT-LP group and RPP-LP group were fewer, whereas the RUT-RPP-LP group elicited the most pronounced apoptotic response. The result demonstrated that the combination of RUT and RES could synergistically enhance tumor cell apoptosis, thereby reinforcing the therapeutic superiority of RUT-RPP-LP for cancer intervention.

#### 4.7.2. CRT, HMGB1 and PD-L1 Expression Levels

As an early ICD marker, CRT translocated from the endoplasmic reticulum to the cell membrane, thereby facilitating recognition by antigen-presenting cells [34]. In contrast, HMGB1, a late ICD signal, activated the immune response mediated via Toll-like receptors when released from the nucleus [35]. IHC analysis is shown in Figure 4d, and both the CRT and HMGB1 signals appear in brown color. Specifically, in the results of CRT staining, pronounced brown staining (indicative of CRT exposure) was observed predominantly at the plasma membrane in the RPP-LP and RUT-RPP-LP groups, confirming enhanced CRT translocation; the staining of HMGB1 revealed that in the normal saline, free RUT + RES, and RUT-LP groups, brown staining largely co-localized with nuclear (blue) signals, suggesting intracellular retention of HMGB1. Conversely, robust extracellular brown deposition was evident in the RPP-LP and RUT-RPP-LP groups, consistent with substantial HMGB1 release. Accordingly, a further quantitative analysis of positive cell rates for each indicator (Figure 4e,f) revealed that RUT-RPP-LP significantly enhanced CRT exposure and HMGB1 release. Integrated CRT and HMGB1 immunostaining demonstrated that RUT-RPP-LP induced ICD, and this induction was mechanistically dependent on RPP.

PD-L1 expression directly reflected the ability of tumor immune escape [36]. PD-L1 staining (Figure 4d) demonstrated markedly reduced brown signal intensity in the RUT-LP and RUT-RPP-LP groups relative to all other groups, indicating significant downregulation of PD-L1 expression. This finding aligned with quantitative PD-L1 protein analysis (Figure 4g), confirming that RUT-RPP-LP mediated ICB through PD-L1 downregulation, and that this effect was attributable to RUT.

Consistent with the IHC results, RUT-RPP-LP comprising RUT and RES may synergistically engage both ICB and ICD pathways. Concretely, RUT mediated downregulation of the key tumor immune escape factor PD-L1 to block the immune checkpoint pathway. Concurrently, RES promoted CRT exposure and HMGB1 release, inducing ICD to enhance the sensitivity of tumor cells to PD-L1 blockade. Together, the complementary mechanisms established a reinforcing cycle of relieving immunosuppression and amplifying immune recognition, significantly potentiating the efficacy of tumor immunotherapy [37,38].

#### 4.7.3. The Level of Key Effector Molecules

TNF-α promoted tumor cell apoptosis and facilitated inflammatory infiltration, whereas IFN-γ directly killed tumors via enhancing antigen presentation and activating macrophage function [39,40]. ELISA quantification (Figure 4h,i) revealed significantly elevated serum concentrations of both cytokines across all treatment groups, with the most pronounced increases observed in the RUT-RPP-LP group. This indicated that the coordinated upregulation of RUT and RES could reshape the immunosuppressive TME and potentiate the cytotoxic function of effector T cells. Accordingly, RUT-RPP-LP mediates dual immunomodulation (combining ICB and ICD), robustly stimulating systemic anti-tumor immunity and concurrently augmenting effector T-cell functionality, thereby enabling effective synergistic anti-tumor therapy.

## 5. Conclusions

In summary, this study constructed a novel spatiotemporal hierarchical controlled-release delivery system of LMH, RUT-RPP-LP, to fully exert the immunomodulatory potential of RUT and RES and realize the ICB/CD synergistic treatment of malignant tumors. RUT-RPP-LP exhibits spherical uniformity, excellent dispersion, long-term stability, and pH-responsive release: stable at physiological pH, but releasing drugs in response to the acidic TME and lysosomes. In vivo tumor inhibition studies showed that RUT-RPP-LP had good biocompatibility, efficient tumor targeting, and powerful anti-tumor efficacy. Mechanistically, its therapeutic activity correlated with downregulation of PD-L1 expression on tumor cells, induction of CRT membrane exposure, extracellular release of HMGB1, and enhanced secretion of key immune effector cytokines. Collectively, RUT-RPP-LP enables spatiotemporally coordinated ICB/ICD synergy, enhancing herbal ingredients’ bioavailability while offering a promising combinatorial immunotherapy strategy.

Nevertheless, this study still has certain limitations. Firstly, the causal link between ICD induction and enhanced intratumoral immune cell infiltration remains to be experimentally validated. Secondly, the optimal drug dosage of RUT and RES for maximal synergistic anti-tumor efficacy requires further systematic optimization. Finally, although previous studies of the research team have demonstrated the corresponding capabilities of rutin and resveratrol, RUT-RPP-LP still lacks corresponding in vitro mechanism verification experiments. In addition, the therapeutic effect in male model mice should also be considered in the future to rule out sex interference.

## Data Availability

The original contributions presented in this study are included in the article. Further inquiries can be directed to the corresponding authors.
